# Imaging Electric Polarization Switching in Multilayer Graphene

**DOI:** 10.1002/advs.75781

**Published:** 2026-05-22

**Authors:** Zhou Zhou, Xiyao Peng, Jianfeng Bi, Jing He, Fei Xue, Jie Jiang, Huizhen Wu, Zhiwen Shi, Haoliang Qian, Toshikaze Kariyado, Sihan Zhao

**Affiliations:** ^1^ School of Physics Zhejiang Key Laboratory of Micro‐Nano Quantum Chips and Quantum Control and State Key Laboratory of Silicon and Advanced Semiconductor Materials Zhejiang University Hangzhou China; ^2^ State Key Laboratory of Chemical Engineering and Low‐Carbon Technology College of Chemical and Biological Engineering Zhejiang University Hangzhou China; ^3^ School of Materials Science and Engineering Zhejiang University Hangzhou China; ^4^ School of Physics and Astronomy and Tsung‐Dao Lee Institute Key Laboratory of Artificial Structures and Quantum Control (Ministry of Education) Shanghai Jiao Tong University Shanghai China; ^5^ State Key Laboratory of Extreme Photonics and Instrumentation ZJU‐Hangzhou Global Scientific and Technological Innovation Center College of Information Science and Electronic Engineering Zhejiang University Hangzhou China; ^6^ Research Center for Materials Nanoarchitectonics (MANA) National Institute for Materials Science (NIMS) Japan

**Keywords:** electric polarization, multilayer graphene, polarization switching, scanning near‐field optical microscopy

## Abstract

Fundamentally distinct from the conventional ferroelectrics, the most discriminative hallmark of the emerging 2D sliding ferroelectricity is the polarization switching via the domain wall (DW) sliding. Meanwhile, multilayer graphene has recently attracted immense research attention owing to engineerable strong electron correlation and non‐trivial band topology. Here, domain wall sliding‐induced electric polarization switching is directly observed for the first time in multilayer graphene. We identify adjacent polar domains of opposite electric polarizations in tetralayer graphene, the thinnest natural graphene polytype with broken inversion and mirror symmetries, by a gate‐tunable nanoscale optical imaging technique. Remarkably, we directly observe and realize the DW sliding‐induced polarization switching between these polar domains upon application of global and local electric fields and mechanical forces. Our combined experiment and theory find a single DW sliding at the middlemost interface is responsible for the polarization switching. Our work opens new opportunities in studying sliding ferroelectricity in multilayer graphene, and demonstrates a novel optical readout method for sensitively and directly detecting electric polarization in 2D materials.

## Graphical abstract

Domain wall sliding‐induced electric polarization switching is directly observed for the first time in multilayer graphene, enabled by a novel optical readout method. The polarization switching is realized upon application of global and local electric fields, and mechanical forces between neighboring polar domains of opposite out‐of‐plane polarizations in tetralayer graphene, via a single DW sliding at the middlemost interface.

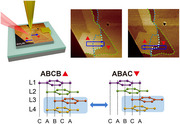

## 1 Introduction

Recent advancements in 2D van der Waals (vdW) materials have unlocked tremendous new opportunities to explore and engineer ferroelectricity at the nanoscale through a new type of ferroelectricity referred to as 2D sliding ferroelectricity [1]. Unlike the ion movement‐induced dipole switching in conventional ferroelectrics, the interfacial incommensurate domain wall (DW) sliding unconventionally couples with the (out‐of‐plane) electric polarizations in commensurate stacking orders in 2D sliding ferroelectricity, which can lead to efficient, robust, and ultrafast polarization switching, on par with that in some of the best conventional ferroelectric materials [2, 3, 4, 5, 6, 7, 8]. 2D sliding ferroelectricity has been extensively studied, through both theory and experiments, in a variety of insulating and/or semiconducting 2D vdW materials and their marginally twisted 2D moiré superlattices, such as hexagonal boron nitride (hBN) [2, 9, 10, 11], MoS_2_ [12, 13, 14, 15, 16, 17], WSe_2_ [3, 12, 14, 18], and InSe [4, 19], where DW sliding at vdW interfaces plays a pivotal role.

Multilayer graphene and their moiré superlattices have recently received tremendous research attention. There, engineerable electron correlation intertwined with non‐trivial band topology has enabled the discovery of exotic quantum states that are gate tunable. These new quantum states include but are not limited to integer and fractional quantum anomalous Hall [20, 21, 22, 23], topological charge density wave [24], signatures of anomalous Hall crystals [25], and new superconductivity [26, 27]. Multilayer graphene‐hBN moiré superlattices were recently detected to show a pronounced current hysteresis when gated [28, 29, 30, 31, 32], a signature that was attributed to sliding ferroelectricity. But the microscopic origin of this effect is not fully resolved due to a lack of direct visualization of the switching. Rich stacking orders in moiréless graphene polytypes offer an alternative opportunity to explore the electric polarization switching phenomena [33, 34, 35, 36, 37, 38]. Recent identification of polar domains in tetralayer graphene by Kelvin Probe Force Microscopy (KPFM) [39] further motivates us to explore the polarization switching phenomena in natural multilayer graphene polytypes.

Herein, we report the first direct visualization of electric polarization switching in tetralayer graphene, the thinnest natural multilayer graphene that hosts intrinsic electric polarization. Our gate‐tunable scanning near‐field optical microscopy (SNOM), clearly identifies the robust electric polarization in adjacent ABAC and ABCB polar domains of opposite electric polarizations even at high carrier density (∼ 5 × 10^12^ cm^−2^). Upon electric fields and mechanical forces, the polarization switching is triggered and accomplished via local DW sliding, which is directly imaged by our gate‐tunable SNOM. The observed microscopic switching process and mechanism in tetralayer graphene are further consolidated by the combined KPFM, Raman spectroscopy, and density functional theory (DFT) calculations. Our work opens new opportunities in studying sliding ferroelectricity in multilayer graphene, and demonstrates a novel optical readout method for sensitively and directly detecting electric polarization in 2D nonpolar materials.

## 2 Results and Discussion

### 2.1 Electric Polarization in Tetralayer Graphene

**FIGURE 1:**
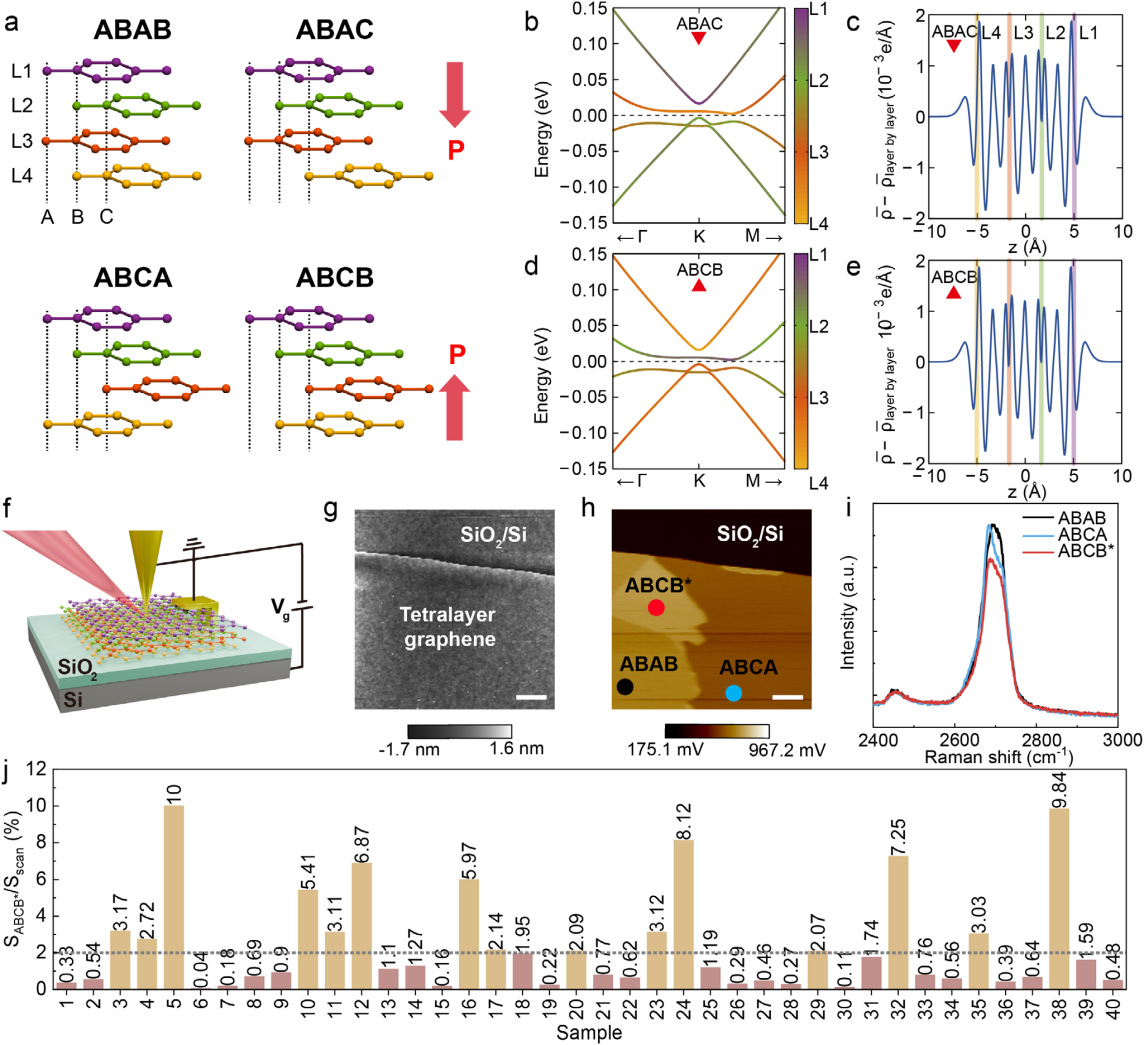
Characterizations of tetralayer graphene with broken symmetries. (a) Stacking configurations of tetralayer graphene. (b, d) Layer‐projected band structure for ABAC and ABCB stackings, respectively. (c, e) Layer‐resolved charge density distribution for ABAC and ABCB stackings, respectively. (f) Schematic of the gate‐tunable SNOM measurement. (g–i) Topography (g), SNOM image (h), and Raman spectra (i) of a typical tetralayer graphene having a polar ABCB‐type (ABCB^*^) stacking order. (j) Statistics of the areal portion for the ABCB‐type domains in this study. The scale bars in g and h are 2 µm.

Bernal stacking (or ABAB) and rhombohedral stacking (or ABCA) are centrosymmetric (Figure 1a), whereas, on the other hand, the ABAC and ABCB stackings with purely stacking‐induced broken (out‐of‐plane) inversion and mirror symmetries carry intrinsic (out‐of‐plane) electric polarization (Figure 1a). This is supported by our DFT calculation of layer‐projected band structure (Figure 1b for ABAC, Figure 1d for ABCB) and charge density distribution (Figure 1c for ABAC, Figure 1e for ABCB), which shows a small bandgap for both polar stackings and that polarization direction pointing downward (upward) for ABAC (ABCB) stacking (Computational details in Section 4 and Note S1). For the latter aspect, we find that more holes reside in L3 (L2) than in L2 (L3) for ABAC (ABCB) and that it exhibits the greatest charge imbalance between the middle two layers, L2 and L3, for both stackings (Figure 1c,e, calculated partial Löwdin charge in Figure S1, and Computational details in Section 4).

We use gate‐tunable SNOM (schematic shown in Figure 1f) to identify different stacking orders in tetralayer graphene that is prepared on a 285 nm SiO_2_/Si substrate (details in Section 4). It was used to directly characterize and distinguish the local plasmonic responses of multilayer graphene with distinct stacking orders at far infrared due to their difference in band structure and local optical conductivity [40, 41, 42]. It was also recently utilized to indirectly probe the out‐of‐plane polarizations of twisted WSe_2_ and hBN moiré superlattices by using graphene as a sensor [18, 43]. One of the advantages of SNOM over techniques such as KPFM is that no sample grounding is needed (if not gating), making SNOM a high‐throughput scanning probe technique in identifying scarce polar domains. Figure 1g,h shows the atomic force microscopy (AFM) topography and SNOM image of a typical tetralayer graphene, where three regions with the same thickness but distinct near‐field optical contrasts are detected.

The three regions in Figure 1h are individual domains of nonpolar ABAB (black dot) and ABCA (blue dot), and a polar ABCB‐type domain (denoted as ABCB^*^ with red dot if not specifically distinguished). This stacking assignment is supported by Raman spectroscopy measurements on the 2D peaks (Figure 1i) [44, 45], being consistent with previous literatures [46, 47, 48]. The 2D peak of the ABAB stacking (black curve in Figure 1i) has the most symmetric spectral shape, whereas that of the ABCA stacking (blue curve in Figure 1i) has the most asymmetric spectral shape, with a strong peak feature on the lower wavenumber side. The spectral shape of the 2D peak of the polar stacking (red curve in Figure 1i) lies between those of the blue and black curves. We note that, however, the 2D peaks in Raman spectra cannot distinguish the two polar stackings [38, 39]. The formation of the observed domain structure is likely related to the unintentional doping, strain, and or pinning from the substrate and the shear strain induced during the exfoliation. We statistically find in this study that 40 out of 132 tetralayer graphene flakes have shown ABCB‐type domains, 15 of which have ABCB‐type areal proportion larger than 2% of the total flake (Figure 1j). More SNOM images of ABCB‐type domains are shown in Figure S2.

### 2.2 Gate‐Tunable SNOM Imaging of Adjacent Polar States with Opposite Polarizations

**FIGURE 2:**
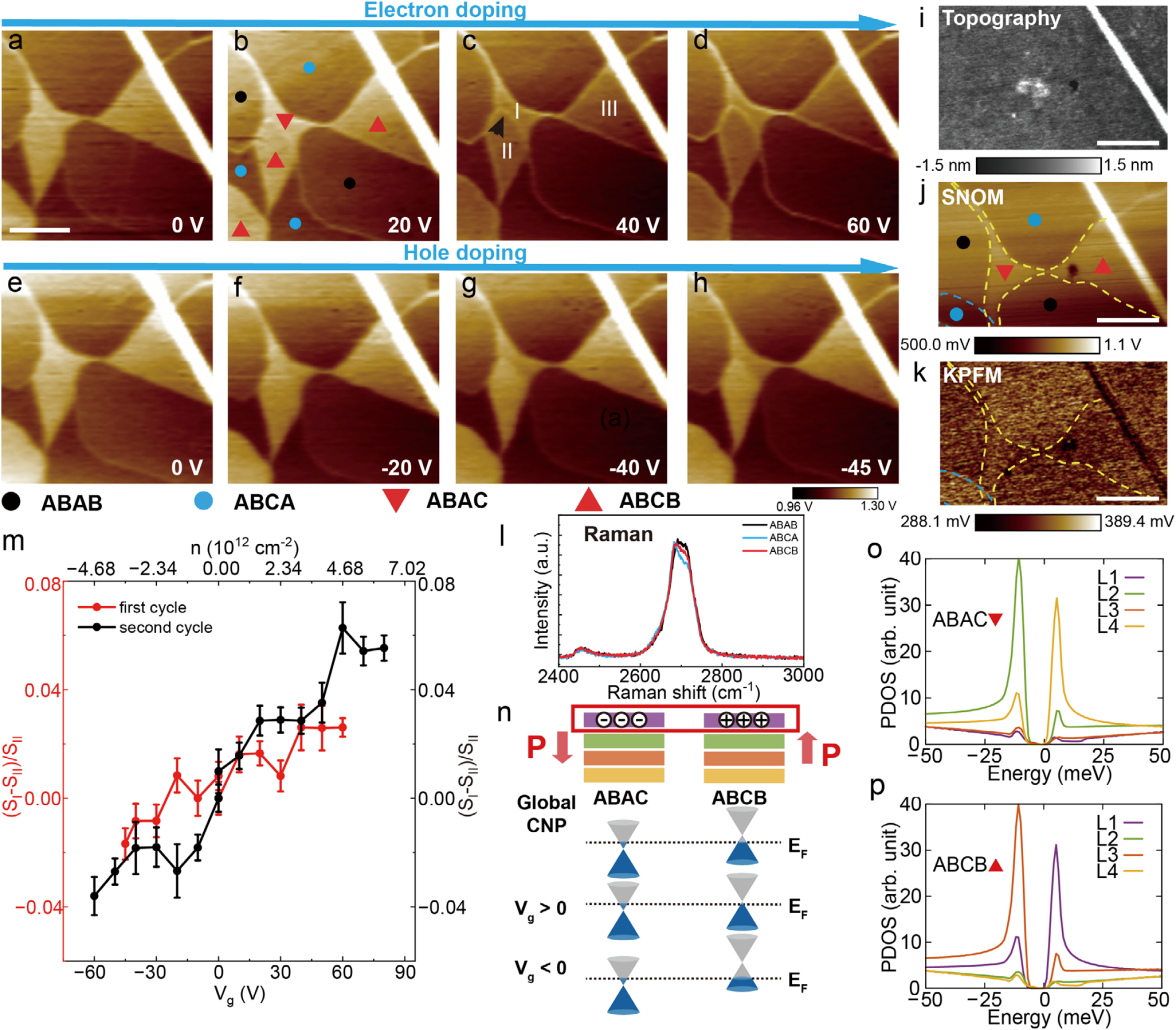
Gate‐tunable SNOM imaging of adjacent polar domains with opposite polarizations (device 1). (a–h) Gate‐dependent SNOM images. Region I is ABAC stacking, and regions II and III are ABCB stackings. (i–k) Topography (i), SNOM (j), and KPFM mapping (k) of device 1 measured at *V_g_* =  0 *V*. The dashed lines depict the contours of the DWs separating different stacking domains. (l) Raman spectra obtained from the three different stackings of the same area (also see Figure S7). (m) Extracted optical contrast between two polar stackings as a function of *V_g_* (bottom axis) and carrier density (*n*, top axis). (n) Schematic drawing to explain the observed optical contrast change in m. (o, p) Calculated PDOS for ABAC and ABCB stackings at CNP, respectively. All the scale bars are 1 µm.

To discern adjacent polar domains with opposite electric polarizations, we apply back‐gate voltages (*V_g_*) to induce electron and/or hole doping in tetralayer graphene while keeping both the tetralayer graphene and the scanning AFM tip grounded (Figure 1f) (details in Section 4). A closed gating loop was obeyed in our gate‐dependent SNOM experiments: *V_g_* sweeps from 0 V to the positive maximum, then to the negative maximum passing through 0 V, and then returns back to 0 V. Figure 2a–h (first cycle scan) present the gate‐dependent SNOM images of a tetralayer graphene device (device 1), in which data measured from *V_g_* = 0 V to *V_g_* = 60 V (positive maximum) and from 0 V to *V_g_* = −45 V (negative maximum) are shown. At *V_g_* = 0 V that is close to the charge neutrality point (CNP) (Figure 2a), SNOM can distinguish Bernal (ABAB), rhombohedral (ABCA), and polar (ABCB^*^) stackings as demonstrated in Figure 1.

For nonpolar stackings, the gate‐dependent SNOM response has been systematically studied [49, 50]. Here, we focus on the polar stacking region in the middle of each SNOM image (marked by triangles in Figure 2b and I, II, and III in Figure 2c). As *V_g_* is increased from 0 to 20 V, a DW across the whole region becomes clearly visible, dividing the original region with nearly identical optical contrast into two parts, region I and region II, with slightly different optical contrasts, as shown in Figure 2b. This DW becomes more apparent as *V_g_* increases to 40 V (Figure 2c and indicated by the black arrow) [51, 52]. As *V_g_* is continuously increased from *V_g_* = 20 V, the near‐field response of region II becomes progressively darker than that of region I (Figure 2b–d). The near‐field response almost returns to its initial state (Figure 2a) when *V_g_* passes through 0 V from the positive maximum, making regions I and II barely distinguishable (Figure 2e). When *V_g_* is gradually changed from *V_g_* = 0 V (Figure 2e) to more negative values (Figure 2f–h), the near‐field response of region I becomes progressively darker than that of region II, an opposite trend to that shown in Figure 2a–d with positive *V_g_*. The complete dataset for the first cycle scan is shown in Figure S3. Reproducible results are observed in a consecutive SNOM measurement (second cycle scan) as shown in Figure S4, with extension to a larger range of *V_g_*, from ‐60 to 80 V.

To quantify the distinct near‐field behavior observed in regions I and II, we extract their optical contrast, which is defined as (*S_I_* − *S_II_*)/*S_II_*, at each fixed *V_g_*, where *S* represents the measured near‐field signal averaged over selected areas in each region (see Section 4 for details). The results for the first and second cycles are shown by the red and black dots in Figure 2m, respectively. We note that region II changes into a negligibly small area due to DW sliding‐induced polarization switching after the first cycle scan, and thus, we present  (*S_I_* − *S_III_*)/*S_III_* for the second cycle scan, since regions II and III have the same stacking. Figure 2m shows that both (*S_I_* − *S_II_*)/*S_II_* and (*S_I_* − *S_III_*)/*S_III_* reverse signs when swapping the carrier type, and that their absolute magnitude increases with carrier doping. Figures S5 and S6 display the full dataset for the extracted optical contrast and confirm its robustness with varying areas for data averaging.

The systematic evolution of the gate‐dependent near‐field response observed in Figure 2a–h and Figure 2m suggests the existence of two distinct polar stackings hosting opposite vertical electric polarizations. Our optical detection of two adjacent polar stackings with opposite vertical electric polarizations is unambiguously verified by KPFM mapping of the same area, as shown in Figure 2k (after second cycle scan and without applying *V_g_*); the corresponding topography and SNOM images are presented in Figure 2i,j, respectively, for comparison [39]. Figure 2l and Figure S7 present the corresponding Raman spectra [44, 45]. Distinct work functions are observed between two adjacent polar stacking orders, where the measured work function of region I is smaller (brighter in Figure 2k) than that of region III. Therefore, we conclude that region I corresponds to ABAC stacking with downward polarization and that region III (also region II) with a larger work function, corresponds to ABCB stacking with upward polarization. These results are consistent with our DFT calculations (Figure 1c,e, and Figure S1). We emphasize that the observed optical contrast difference between two opposite polar stackings sustains at least up to a carrier density of $∼5\times 10^{12}\;cm^{-2}$ (Figure 2m), larger than that implied by a recent KPFM study [39]. This experimental observation clearly indicates the coexistence of electric polarization and a large number of free carriers in 2D multilayer graphene with broken symmetries. We also systematically measure three extra devices (devices 2, 3, and 4) via gate‐dependent SNOM, KPFM, and Raman spectroscopy, obtaining consistent results as observed in device 1 (from Figure S8–S16).

The electric polarization in multilayer graphene is predicted to be extremely weak, at least one to two orders of magnitude smaller than vdW polar materials [34]. Our result thus also establishes SNOM as the first optical technique capable of directly and sensitively probing symmetry‐breaking polar states in 2D multilayer graphene. The optical detection of polar domains with opposite polarization directions (2a–m) can be qualitatively explained as follows. We assume that the detected near‐field signal underneath the metallic AFM tip is predominantly contributed by the free carriers residing in the topmost graphene layer [40, 41, 42]. At *V_g_* = 0 V (CNP), there are equal electrons and holes in the topmost graphene layer in ABAC and ABCB, respectively, due to the purely stacking‐induced broken symmetries (obtained from DFT calculation in Figure S1, and a schematic is shown in Figure 2n) [18, 43]. When *V_g_* > 0, doped electrons in ABAC are mainly filled into the bottom layer L4, and virtually no electrons are filled into the top layer L1 according to the calculated layer‐projected density of states (PDOS) shown in Figure 2o. As a result, the carrier concentration in the topmost layer L1 basically remains unchanged in ABAC (Figure 2n). On the other hand, most of the doped electrons in ABCB are filled in the topmost layer (L1) according to the calculated PDOS (Figure 2p) when *V_g_* > 0, resulting in decreased carrier concentration in L1 (Figure 2n). The above explains why ABCB becomes increasingly darker than ABAC with increased electron doping (from Figure 2a–d). The trend upon hole doping (*V_g_* < 0) can be similarly understood, which is illustrated in Figure 2n.

### 2.3 Polarization Switching in Gated Tetralayer Graphene

**FIGURE 3:**
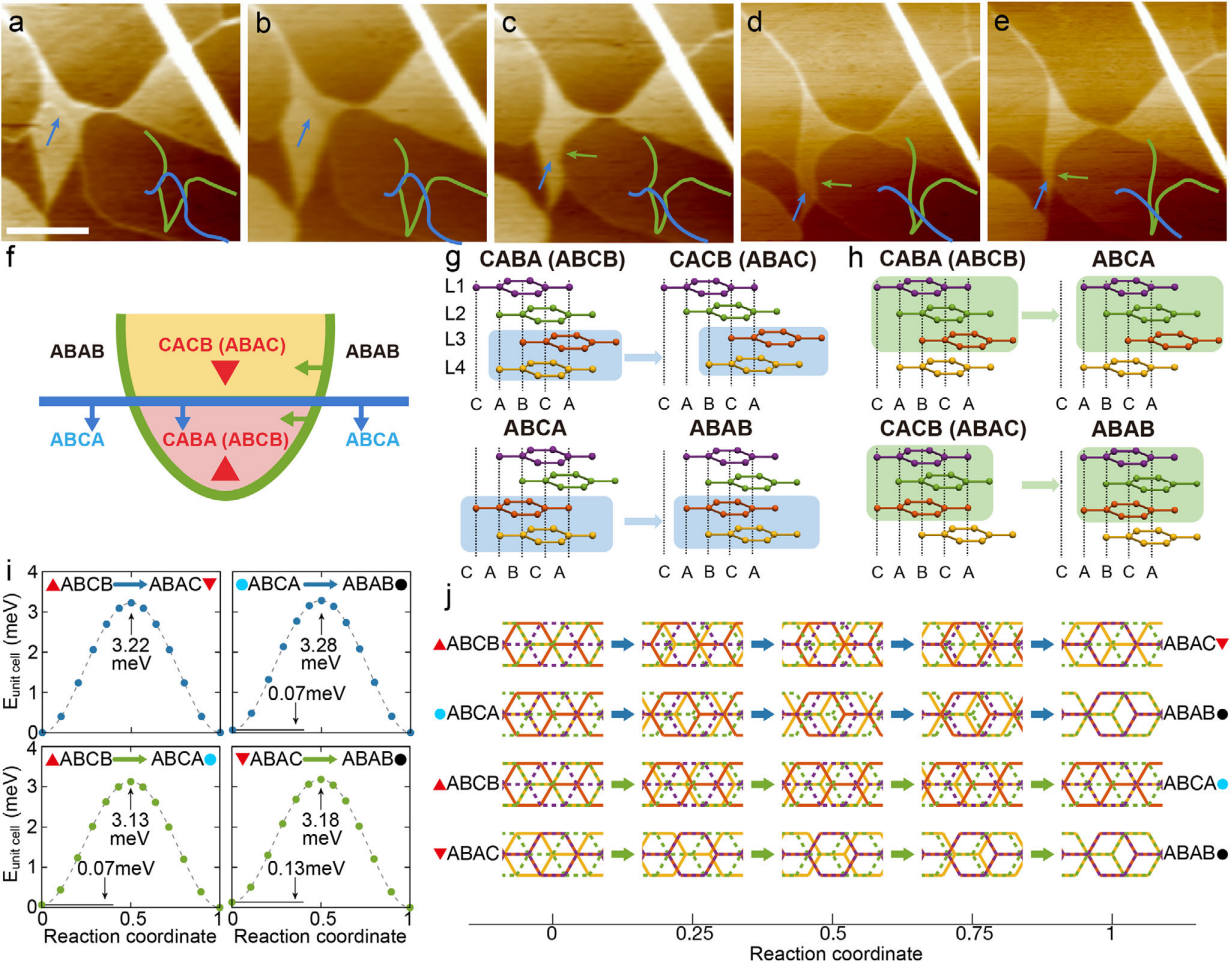
Polarization switching in tetralayer graphene by a global gating (device 1). (a–e) SNOM images capturing the sliding motions of two different types of DWs confined at different interfaces (indicated by blue and green arrows and solid contours). Microscopic polarization switching process from ABCB to ABAC is observed via a DW (blue arrows and contours) sliding mechanism. (f) Schematic reconstruction of domain patterns and DW sliding motions observed in a to e. (g, h) Schematic illustration of interlayer sliding induced by blue and green DW sliding motions, respectively. (i) Calculated total energies for the four switching pathways observed in a to e, with the upper and lower two panels corresponding to situations in g and h, respectively. Each point in i denotes a state of crystalline structures along the reaction path. (j) The crystalline structures of initial, intermediate, and final states on the four reaction paths shown in i. Layers from L1 to L4 are encoded in colors (dashed lines for L1 and L2). The scale bar in a is 1 µm.

**FIGURE 4:**
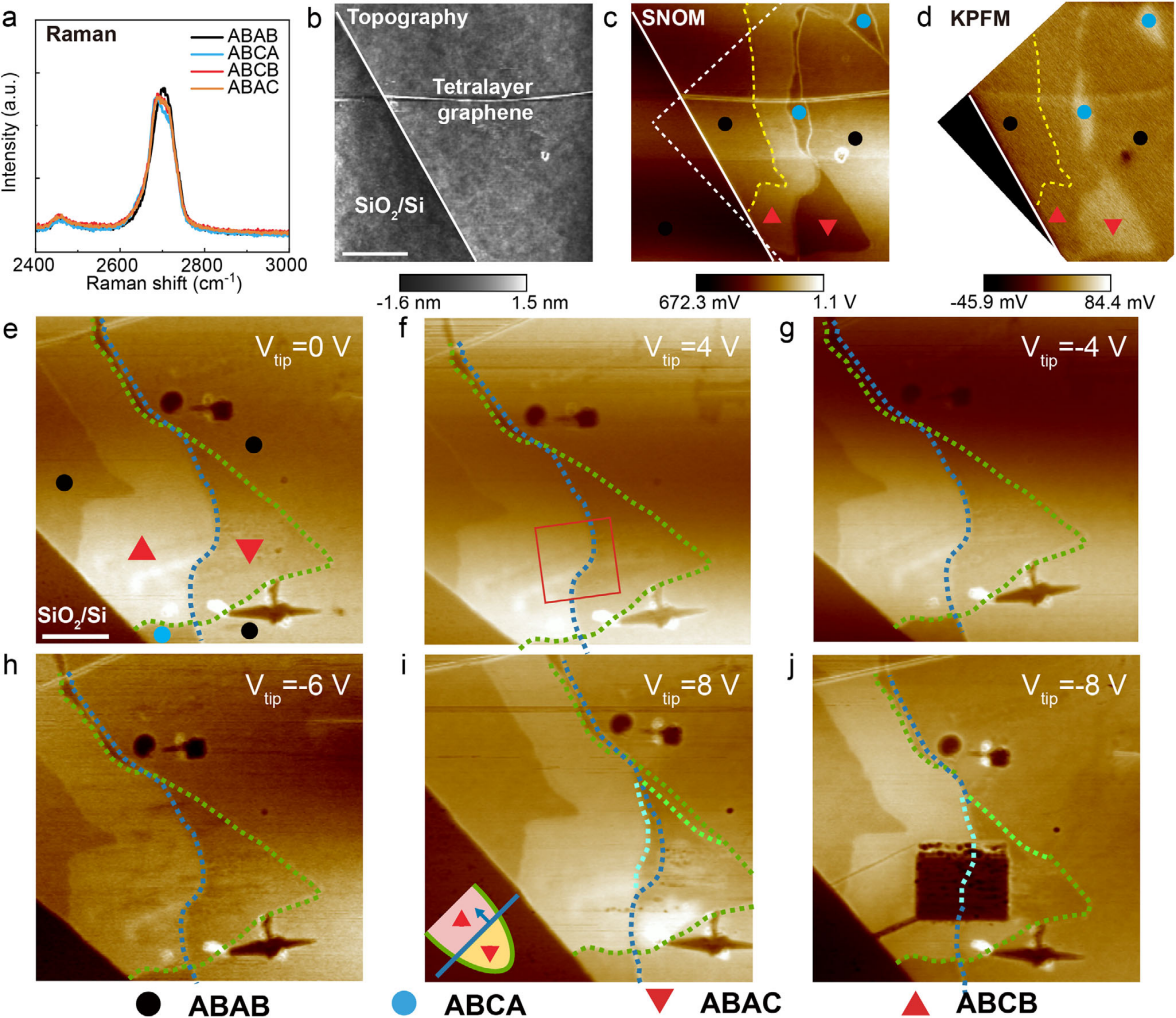
Polarization switching in tetralayer graphene by a local electric field (device 2). (a) Raman spectra of device 2 for the four different stackings (also see Figure S9). (b–d) Topography, SNOM, and KPFM mapping of the polar stackings at *V_g_* =  0 *V*. The yellow dashed lines in c and d depict the DW contours separating ABCB and ABAB. (e–j) SNOM images after scanning with *V_tip_* =  0, 4, − 4, − 6, 8, and − 8 *V*, respectively, within the area of the red box in f. At *V_tip_* =  8 *V* (i), DW between two polar states moves from its original position (blue dashed line) to a new position (cyan dashed line), transforming ABCB to ABAC. The other DW also moves from its original position (green dashed line) to a new position (light green dashed line). Reverse polarization switching is not observed at *V_tip_* =   − 8 *V*. The scale bars in b and e are 2 and 1 µm, respectively.

Figure 3a–e displays representative SNOM snapshots of device 1, capturing the microscopic polarization switching under a global gating condition. Figure 3a–c is obtained in the first cycle and Figure 3d,e is obtained in the second cycle, respectively (also see Figures S3 and S4). We identify two distinct types of DWs and their motions, which are indicated by blue and green contours and arrows in Figure 3a–e. In Figure 3f, we reconstruct the domain patterns and DWs separating and shared by these domains from Figure 3a–e, with the observed DW moving directions indicated by the arrows. A key observation is the polarization switching from ABCB (polarization upward) to ABAC (polarization downward) via a single DW sliding motion indicated by the blue arrows and contours (Figure 3a–f). This microscopic observation, for the first time, unambiguously demonstrates the polarization switching phenomena in multilayer graphene and the underlying DW sliding mechanism responsible for the polarization switching. We note that the observed polarization switching from ABCB to ABAC is observed under a global negative *V_g_* (Figure S3r, after *V_g_* = ‐40 V). This means a finite electric field pointing downward switches the ABCB domain with an upward dipole to the ABAC domain with a downward dipole. This is an energetically favorable process. We note that the reverse switching process (from ABAC to ABCB) is not observed at positive *V_g_*. This is similarly observed in the switching experiment by a local electric field of device 2 (to be shown in Figure 4). The downward motion of the blue DW in Figure 3f also simultaneously expands ABAB at the cost of ABCA.

Interlayer sliding accompanies the in‐plane DW sliding within the region confined by the DW's initial and final positions, giving rise to a change of the local stacking configuration as the DW slides. Considering the shortest sliding paths and the fewest involved interfaces, the movement of the blue DW in Figure 3f can only be achieved by either sliding of the bottom two layers with the top two layers fixed (as illustrated in Figure 3g) or by sliding of the top two layers with the bottom two layers fixed (as illustrated in Figure S17a). These two scenarios are completely equivalent. In both cases, DW sliding occurs in between the middlemost layers L2 and L3, accompanied by an interlayer sliding locally by a carbon‐carbon distance along the armchair direction (Figure 3g). The above arguments for the blue DW sliding (Figure 3f) are also augmented by our calculated switching pathways shown in Figure 3i,j using the nudged elastic band (NEB) method (see details in Section 4). According to the upper two panels of Figure 3i, although the two sliding pathways have slightly different thermodynamic stabilities (0 and 0.07 meV/unit cell between initial and final states, respectively) [34, 44, 45], the calculated energy barrier heights via these two different pathways are both close to 3.2 meV/unit cell, implying that there is little preference between the two pathways in terms of the energy barrier to surmount. In addition, our calculated pathways along which the blue DW slides confirm that the interlayer sliding happens between the top two and bottom two graphene layers along the armchair direction, with a local in‐plane lattice shift by a carbon–carbon bond length. This can be clearly seen from the upper two rows of Figure 3j, where the initial, intermediate, and final states are presented along each the reaction coordinate ranging from 0 to 1. Therefore, we conclude that the polarization switching in tetralayer graphene is continuous sliding via a single DW (blue DW in Figure 3f) that is confined at its middlemost interface.

The other type DW motion indicated by the green arrows and contours in Figure 3a–f is confined at a different interface in comparison to the blue DW (Figure 3a–f). It corresponds to the sliding of the top three layers with respect to the bottom layer, with one possible way illustrated in Figure 3h and the other in Figure S17b. The lower two panels of Figure 3i shows that two sliding pathways involved in the green DW sliding also have similar barrier heights as those involved in the blue DW sliding (upper two panels of Figure 3i). The lower two rows of Figure 3j present the corresponding initial, intermediate, and final states of two pathways shown at the lower two panels of Figure 3i.

### 2.4 Polarization Switching by a Local Electrical Gating

**FIGURE 5:**
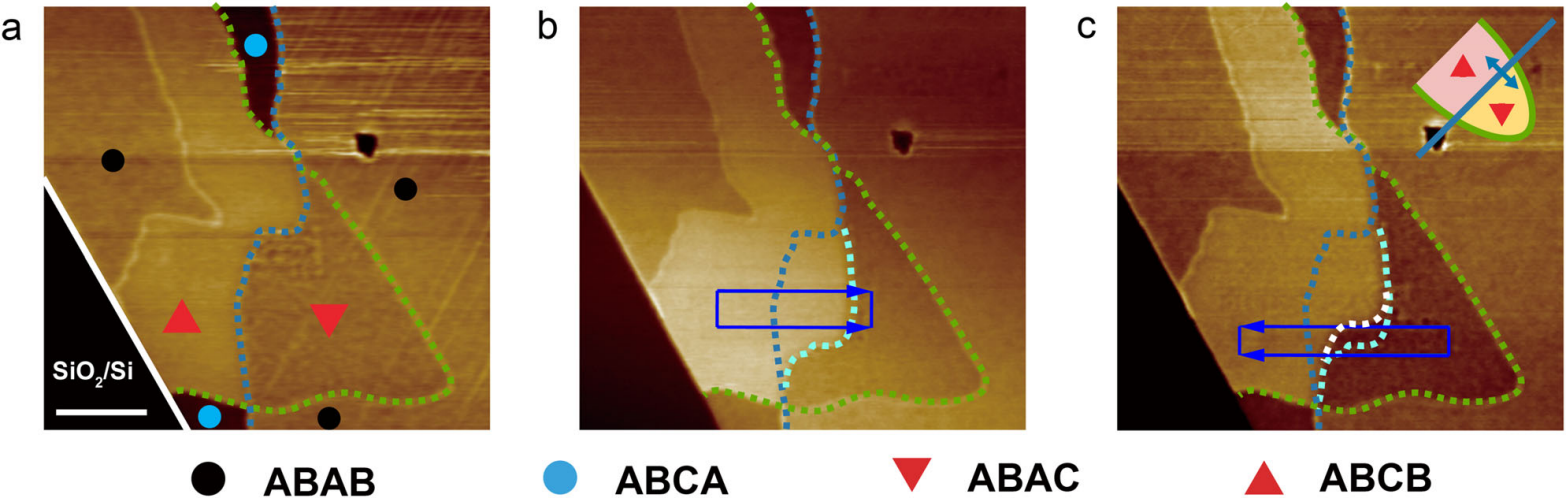
Reversible polarization switching in tetralayer graphene by mechanical forces (device 2). (a–c) SNOM images before tip mechanical manipulation, after tip dragging from left to right, and after tip dragging from right to left, respectively. In b, DW is moved from the blue dashed line to the cyan dashed line. In c, DW is moved back from the cyan dashed line to the white dashed line. The blue rectangles in b and c mark the areas with tip dragging, with arrows indicating the tip moving directions. The scale bar in a is 1 µm.

Under the global gating (Figure 3), the electric field is uniformly applied across the multiple polar and nonpolar domains separated by DWs. We also realize a polarization switching in device 2 by applying a local electric field between the biased tip (*V_tip_*) and the grounded sample, in the vicinity of the DW that separates two adjacent polar domains of opposite polarity (see details in Section 4) [10]. Figure 4a–d shows the Raman spectra, topography, SNOM, and KPFM characterizations of device 2, which shows identical domain patterns and DW types as observed in device 1 (Figure 3). Similar to device 1, we can identify two adjacent polar ABAC and ABCB stackings from the near‐field optical response as shown in Figure 4c, but without gating since this sample is unintentionally hole‐doped after mechanical exfoliation (see Figure S8 for the gate‐dependent SNOM data). Figure 4e is a zoomed‐in image of Figure 4c obtained after the mechanical manipulation (after Figure 5c), but prior to the application of a local electric field (*V_tip_* =  0 *V*). Two types of DWs are depicted by the dashed blue and green lines. We scan the area within the red box of Figure 4f by fixing a certain *V_tip_* at a time, and examine the domain change by SNOM with a grounded tip after it. Figure 4f–j presents the resultant SNOM images with sequentially applying *V_tip_* =  4  − 4, − 6, 8, and − 8 *V*, respectively. The DW between two polar states (blue dashed line) remains unchanged until *V_tip_* =  8 *V* is applied (Figure 4i), transforming ABCB with upward polarization to ABAC stacking with downward polarization. The cyan dashed line in Figure 4i marks the new DW position. This observation means that the localized electric field pointing downward (positive *V_tip_*) switches the upward dipole in ABCB to the downward dipole in ABAC, via a DW sliding mechanism. This is also an energetically favorable process. We note that the other DW (indicated by the green dashed line) also slightly moves, converting the ABAC domain with downward polarization to the nonpolar ABAB domain (new DW position is marked by the light green dashed line in Figure 4i). However, a reverse switching process between two polar domains is not observed when the tip bias is set to *V_tip_* =   − 8 *V* (Figure 4j). Instead, the area after scanning shows a dark contrast. The absence of the reverse switching process is also observed in device 1 under a global gating condition (Figure 3). We do not fully understand this switching asymmetry yet. A recent theoretical work suggests that the underlying SiO_2_/Si substrate (as used in our experiment) affects the electric potential of the bottom graphene layer, causing a finite difference in the absolute dipole strength and thus external switching electric field for two polar stacking in tetralayer graphene [53]. Future experiments on samples encapsulated within two hBN flakes should help to alleviate the substrate effect.

### 2.5 Polarization Switching by Mechanical Tip Motion

Finally, we realize a reversible polarization switching by mechanically applying a force with an AFM tip to directly drag the DW soliton (Section 4) [54]. Figure 5a shows a SNOM image of device 2 prior to tip manipulation. The blue rectangles and arrows in Figure 5b,c mark the tip scanning areas and the tip movement directions during mechanical manipulation, respectively. After the sample is scanned from left to right (Figure 5b), the DW between two polar states is pulled to the right, transforming the ABAC to ABCB (new DW position indicated by the cyan dashed line). Figure 5c shows the resulting pattern after the tip is scanned from right to left, in which the ABCB is partially transformed back into the ABAC (new DW position indicated by the white dashed line). The DW is not fully returned to its original position, which is presumably caused by the presence of DW pinning points and a slight mismatch between the two scanning areas in Figure 5b,c.

## 3 Conclusion

To summarize, we have shown the first direct imaging of electric polarization switching in multilayer graphene. We demonstrate that both electric fields and mechanical forces can be used to control the switching process in tetralayer graphene, the thinnest natural graphene polytype hosting spontaneous electric polarization. We unambiguously observe the sliding DW mechanism that governs the polarization switching of tetralayer graphene, and identify that this DW slides at its middlemost interface. Our work extends the study of 2D sliding ferroelectricity into multilayer graphene systems. Exploring the interplay between electric polarization and doping, the possibility of an ultrafast optical control of polarization switching, etc. will be intriguing future directions.

## 4 Experimental Section/Methods

### 4.1 Sample Fabrication

The graphene samples were mechanically exfoliated from the bulk graphite crystals (NGS graphite flakes) onto 285 nm SiO_2_/Si substrates (heated at 90°C for 1 min before exfoliation) by using Scotch tapes. The substrates were pre‐treated with O_2_ plasma for 4 mins to improve surface cleanliness and adhesion prior to the mechanical exfoliation. The tetralayer graphene regions were identified based on the combined optical contrasts and AFM heights.

### 4.2 Gate‐Tunable Scanning Near‐Field Optical Microscopy (SNOM)

The SNOM measurements were performed under ambient conditions. An infrared laser at ∼ 10.6 µm was focused onto the apex of the gold‐coated conductive AFM tip, which was operated in tapping mode (∼ 200 kHz) during sample scanning. The signal scattered by the oscillating tip was subsequently detected by a high‐speed HgCdTe detector and demodulated at the third harmonic of the tip tapping frequency by a lock‐in amplifier to suppress the large back‐reflected background. SNOM and topography images were simultaneously obtained. The gate‐dependent SNOM measurements were carried out by following a closed gating loop, that is, we set the *V_g_* sweep from 0 V to the positive maximum, then from the positive maximum to the negative maximum, passing through 0 V, and then returned from the negative maximum back to 0 V.

### 4.3 Raman Spectroscopy

Raman spectra were taken using a 532 nm laser focused by an objective (Nikon, with NA = 0.60). The backscattered signal was collected by the same objective and filtered before being dispersed by a 1200/mm grating spectrometer and detected by a liquid‐N_2_‐cooled silicon detector (Princeton Instruments). The typical laser power and exposure time used were ∼ 1.1 mW and ∼ 60 s. The 2D peaks in graphitic multilayers, arising from a double‐resonance Raman process, are sensitive probes of the local electronic band structure, and their spectral shape is widely used to distinguish different stacking orders in the literature [44, 45, 46]. We note that the 2D peaks in Raman spectra cannot be used to distinguish the two polar stackings [38, 39].

### 4.4 Kelvin Probe Force Microscopy (KPFM)

KPFM measurements were conducted using a commercial scanning probe microscopy system (Dimension Icon, Bruker) in frequency‐modulated (FM)‐KPFM mode. Tips with a mechanical resonance frequency of ∼ 75 kHz and a force constant of ∼ 3 N/m were used. Areas with larger voltages in the KPFM mapping (brighter areas) correspond to stackings with lower work functions. For tetralayer graphene, the ABCA domains are the brightest, corresponding to the lowest work function. For the two nearby polar stackings indicated by downward and upward triangles, the former (downward triangle in Figures 2 and 4 of the main text, Figures S11b and S14b) appears to be brighter than the latter (upward triangle in Figures 2 and 4 of the main text, Figures S11b and S14b). This means that the two stackings have downward and upward polarizations. They are thus determined to be ABAC and ABCB stacking orders, respectively, according to our definition in Figure 1a in the main text.

### 4.5 Near‐Field Optical Contrast Between Polar Domains

To extract the optical contrast between, for example, regions I and II, i.e., (*S_I_* − *S_II_*)/*S_II_*, at each fixed back‐gate voltage, we selected two small square areas in each domain (Figures S6 and S7). We extracted (*S_I_* − *S_II_*)/*S_II_* by using the near‐field signals *S_I_* and *S_II_* averaged over two selected square areas in each domain. The error bar of (*S_I_* − *S_II_*)/*S_II_* at each fixed back‐gate voltage was computed by using the following formula [55]: $u={\left(\frac{S_{I}}{S_{II}}\right)}^{2}\;\cdot \left(\frac{u_{1}^{2}}{S_{II}^{2}}+\frac{u_{2}^{2}}{S_{II}^{2}}\right)$, where *u_I_* and *u_II_* are the standard deviations of *S_I_* and *S_II_*, respectively. The carrier density *n* at a fixed *V_g_* was estimated from the capacitance model $C=\frac{ne}{V_{g}}=\frac{\varepsilon_{r}\varepsilon_{0}}{d}$, where *d*  =  285 nm and ε_*r*_ =  4 are the oxide thickness and dielectric constant, respectively, *e* is the elementary charge, and ε_0_ is the vacuum permittivity.

### 4.6 Electrical Switching of Polarization by a Local Electric Field

To apply a local electric field to switch the polarization, the metallic AFM tip operated in a contact mode was biased with DC voltages while the tetralayer graphene sample was kept grounded. To rule out the possibility that the movement of the domain wall was caused by the tip operated in the contact mode itself, we scanned the same area under the same tip scanning condition without applying bias, and we did not discern any DW movement by the contacted tip itself.

### 4.7 Mechanical Manipulation of DWs with an AFM tip

The AFM tip was operated in normal tapping mode during forward scanning, but it was lifted down by ∼ 0.3 µm (lift mode) during backward scanning with the feedback loop closed. Such scanning with a certain downward lift gave rise to a large lateral force that could slide the DW [56, 57]. By rotating the scan area, we reversed the tip sliding direction. Since the feedback was turned off during backward scanning, the gold on the tip apex was unavoidably worn out. Therefore, the SNOM images shown in Figure of the main text were scanned by using different tips.

### 4.8 Computational Details

Density functional theory (DFT) calculations were performed via the Quantum Espresso package [58, 59]. For structural relaxation, the rev‐vdW‐DF2 functional [60, 61] was used to consider vdW interactions. After the structures were relaxed, the band structures and density of states (DOS) were calculated with the Perdew–Burke–Ernzerhof (PBE)‐generalized gradient approximation (GGA) [62] functional. We adapted pseudopotentials constructed with the projector augmented wave (PAW) method using parameters in PSlibrary [63, 64]. The energy cutoffs of the wavefunctions and charge densities were 80 Ry and 640 Ry, respectively. In self‐consistent DFT calculations, k‐points were sampled on *N* × *N* × 1 regular grids in which the in‐plane grids were shifted by a half grid, and the convergence was checked up to *N*  =  264 (see Note S1 and Figure S18). In the DOS calculations, k‐points were sampled in a small region near the K‐point in the Brillouin zone (a circular region with an area of approximately 0.165% of the Brillouin zone area) to reduce the computational cost. For the charge distribution analysis, we calculated the partial Löwdin charge for each carbon atom (Figure S1). Here, “partial” means that the integration over the Brillouin zone was limited to a small portion, as in the DOS calculations, since the important contributions were from the region near the K‐point. Energy barriers between stacking patterns were estimated using the NEB method with 13 images along the reaction path (see Figure 3j).

## Funding

This work is supported mainly by the National Key R&D Program of China (2023YFA1407900 and 2022YFA1203400), the National Natural Science Foundation of China (92580112, 12174335, 12374292, and 62175217), and the Zhejiang Provincial Natural Science Foundation of China (LR23A040002). TK was supported by JSPS KAKENHI Grant Number JP24K06968. The calculations in this study were performed with the Numerical Materials Simulator at NIMS and using the facilities of the Supercomputer Center, the Institute for Solid State Physics, the University of Tokyo.

## Conflicts of Interest

The authors declare no conflicts of interest

## Supporting information




**Supporting File**: advs75781‐sup‐0001‐SuppMat.pdf.

## Data Availability

The data that support the findings of this study are available from the corresponding author upon reasonable request
