# Supplementary material for: Imaging Electric Polarization Switching in Multilayer Graphene
Source: Adv Sci (Weinh). 2026 May 22;13(36):e75781. doi: 10.1002/advs.75781 (PMC13335981; doi:10.1002/advs.75781)
Supplement: Supplementary file 1 — Supporting File: advs75781‐sup‐0001‐SuppMat.pdf. [file ADVS-13-e75781-s001.pdf]

Supporting Information

Imaging Electric Polarization Switching in Multilayer Graphene

Zhou Zhou†, Xiyao Peng†, Jianfeng Bi, Jing He, Fei Xue, Jie Jiang, Huizhen Wu, Zhiwen Shi, Haoliang Qian, Toshikaze Kariyado*, Sihan Zhao**

Supplementary Note 1.

In the literature, there were found some variations in details of the band structure for ABCB/ABAC stackings. Mostly, the differences are explained by differences in theoretical or computational methods, e.g., a tight-binding model or DFT based calculations. However, already within the DFT, there is subtlety about convergence with respect to the momentum space k-mesh sampling. In practice, $N \times N$ in plane regular grids are used in the calculations. Note that the regular grids can be placed with half-grid shift to avoid the exact Γ-point or without such shift to include the exact Γ-point. Figure S18 shows that the convergence is particularly slow without the half-grid shift, and the band structures look a little different for small k-sampling numbers (e.g., extra band crossing at the conduction band side).

Supplementary Figure 1.

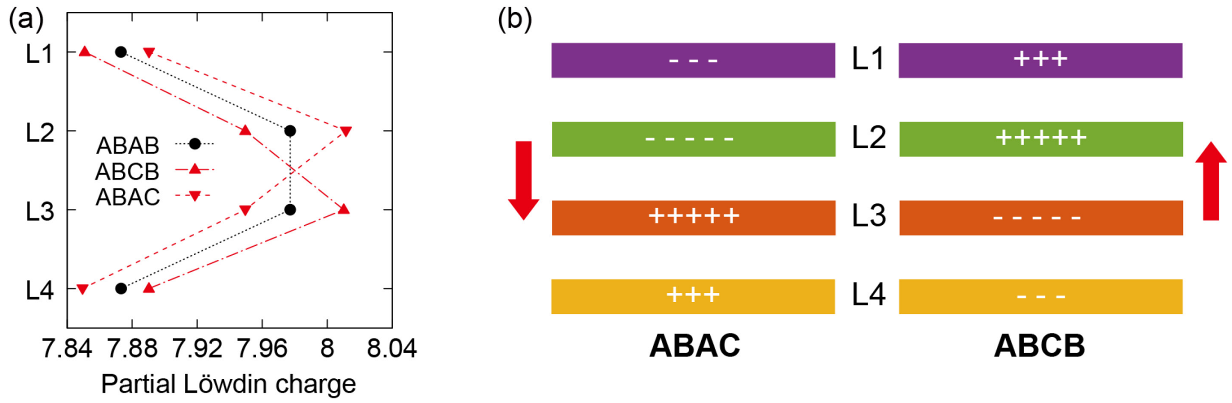

Figure S1. Partial Löwdin charge for each layer in the ABAC and ABCB stackings. (a) Calculated partial Löwdin charge for each carbon atom. Considering the ABAB case as a reference (black dots and dotted line), there are fewer electrons in L1 and L2 and more electrons in L3 and L4 in the ABCB stacking, whereas there are more electrons in L1 and L2 and fewer electrons in L3 and L4 in the ABAC stacking. For both polar stackings, the charge imbalance, in comparison with that for ABAB, is greater in L2 and L3 than in L1 and L4. (b) Schematic drawing of the charge distributions for ABAC and ABCB according to (a).

Supplementary Figure 2.

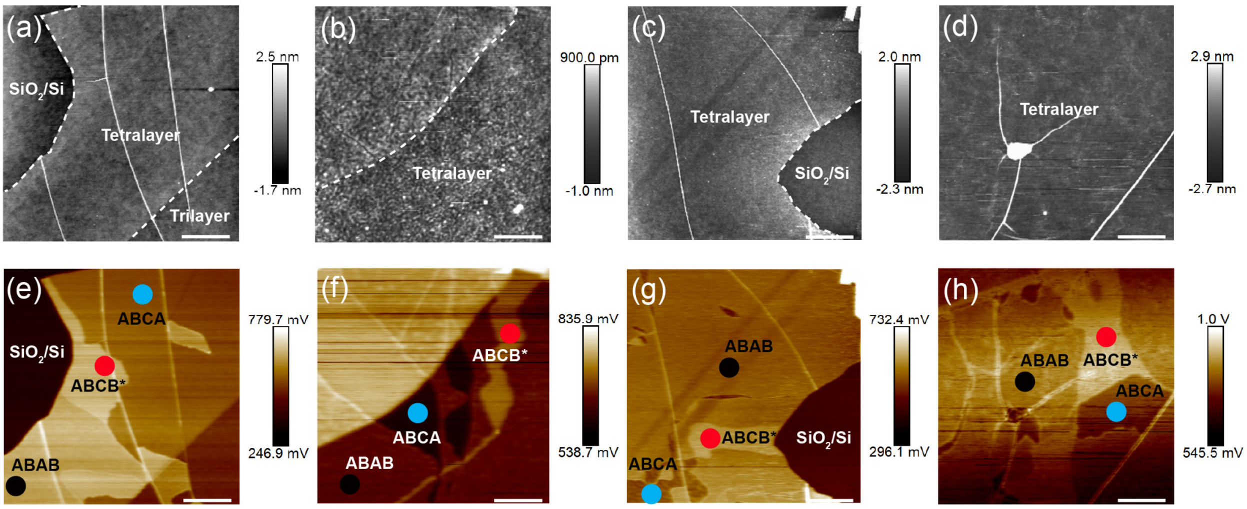

Figure S2. SNOM characterization of tetralayer graphene with nonpolar and polar domains. (a)-(d) Topographies of four representative tetralayer graphene samples. The tetralayer regions are marked by white dashed lines. (e)-(h) SNOM images corresponding to the topographies in (a)-(d). The scale bars in (a) and (e) are 2 μm. The scale bars in (b), (d), (f), and (h) are 1 μm. The scale bars in (c) and (g) are 3 μm.

Supplementary Figure 3.

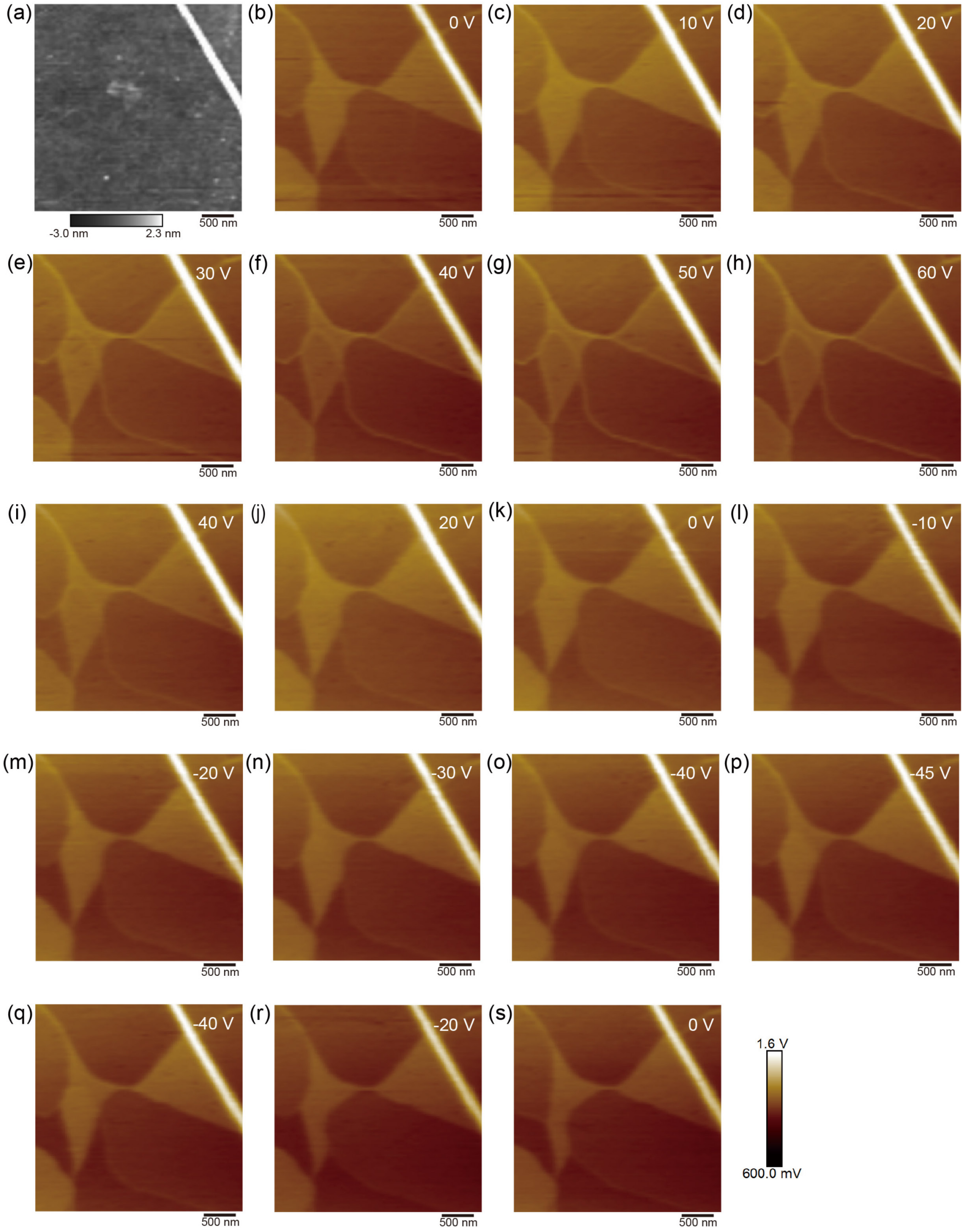

Figure S3. Full SNOM dataset for the first cycle scan of device 1. (a) Topography of device 1. (b)-(s) SNOM images at different V_g values following the applied V_g sequence in our experiment. The applied V_g ranges between -45 V and 60 V. All the presented figures share the same color bar (600 mV-1.6 V). (b), (d), (f), (h), (k), (m), (o), and (p) are presented in Figure 2a-h of the main text. The weak fringe features in the vertical direction of the SNOM images are presumably caused by the interference effect arising from light scattered from the nearby silver paste used to make sample contact. Figure 3a-c in the main text is from this first cycle measurement (Figure S3), and the corresponding figures are (n), (o), and (r).

Supplementary Figure 4.

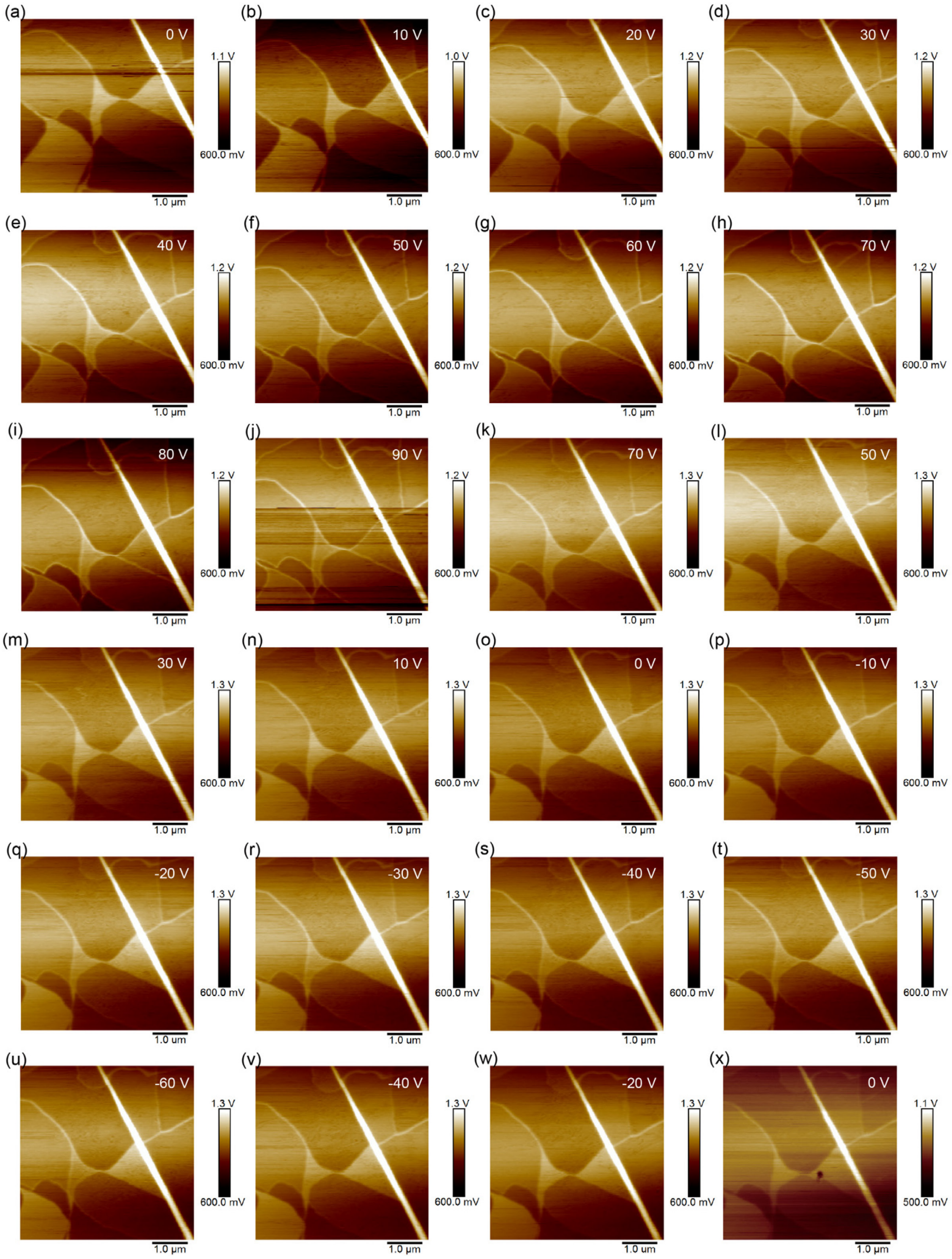

Figure S4. Full SNOM dataset for the second cycle scan of device 1. (a)-(x) SNOM images at different V_g values following the applied V_g sequence in our experiment. The V_g applied ranges between -60 V and 80 V, which is a larger range than that in the first cycle measurement shown in Figure S3. The weak fringe features in the vertical direction of the SNOM images are presumably caused by the interference effect arising from light scattered from the nearby silver paste used to make sample contact. Figure 3d and 3e of the main text are from the second cycle measurement, and they correspond to (l) and (m) of Figure S4, respectively. Both images in the main text share the same color scale bar from 600.0 mV to 1.3 V.

Supplementary Figure 5.

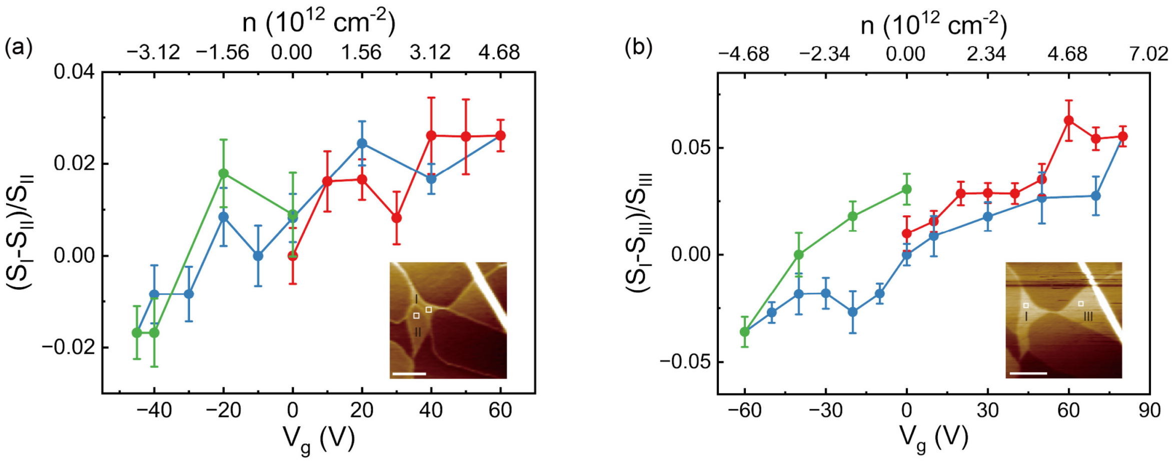

Figure S5. Full dataset for the optical contrast extracted from the two scan cycles of device 1. (a) and (b) Full dataset for the optical contrast between the two polar stackings extracted from the first cycle (a) and second cycle (b) of gate-dependent SNOM measurements. In both (a) and (b), the extracted optical contrasts from $V_g = 0$ V to the positive maximum V_g are denoted by red dots, those measured from the positive maximum V_g to the negative maximum V_g are denoted by blue dots, and those measured from the negative maximum V_g back to $V_g = 0$ V are denoted by green dots. The results shown in Figure 2m of the main text correspond to the data denoted by the red dots and the data denoted by the blue dots on the negative V_g side. Insets in (a) and (b): the white boxes label the sample areas of the two nearby regions from which we extracted the average optical near-field response and obtained the optical contrast. The scale bars in the two insets are both 1 μm.

Supplementary Figure 6.

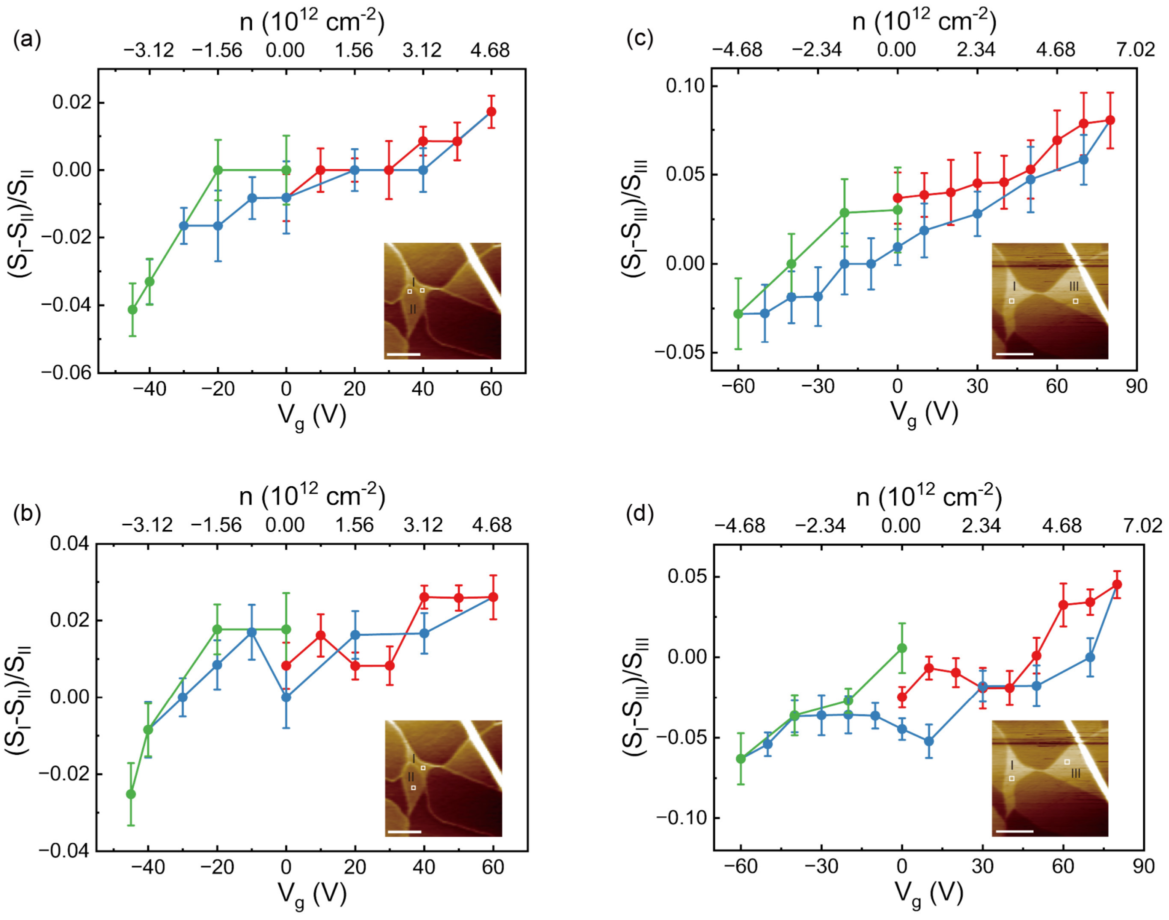

Figure S6. Examination of the extracted optical contrast between two polar stackings of device 1 by selecting different areas. (a)-(d) Optical contrast between two polar stackings extracted along the same scanning rows [(a) from the first cycle and (c) from the second cycle] and different scanning rows [(b) from the first cycle and (d) from the second cycle] for the two cycles gate-dependent SNOM measurement. In (a) to (d), the extracted optical contrasts from V_g = 0 V to positive maximum V_g are denoted by red dots, those measured from positive maximum V_g to negative maximum V_g are denoted by blue dots, and those measured from negative maximum V_g back to V_g = 0 V are denoted by green dots. When comparing (a) with (b) and (c) with (d), we can observe that despite the selected areas are different, the trend of the extracted optical contrast upon varying V_g is the same, consistent with those shown in Figure 2m of the main text. Insets in (a) to (d): the white boxes label the sample areas from which we extract the average optical near-field response and obtain the optical contrast. The scale bars in all the insets are 1 μm.

Supplementary Figure 7.

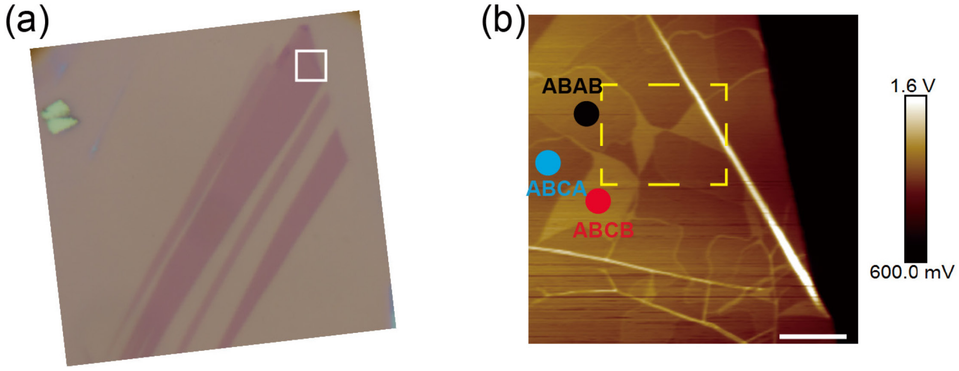

Figure S7. Raman spectra measurement of device 1. (a) Optical image of the tetralayer graphene. (b) SNOM image of the same area shown inside the white box in (a). The scanning area in Figure 2a-h of the main text coincides with the area enclosed by the yellow dashed frame. The Raman spectra in Figure 2l are obtained from the three different stackings, whose measurement points are marked in (b). Note that the small area of polar domains [at the center of the yellow dashed frame in (b)] makes direct far-field Raman measurements challenging. Instead, we measure the Raman spectra of the nearby polar domain with identical optical near-field responses [red dots in (b) with an identified ABCB stacking order]. The scale bar in (b) is 2 μm.

Supplementary Figure 8.

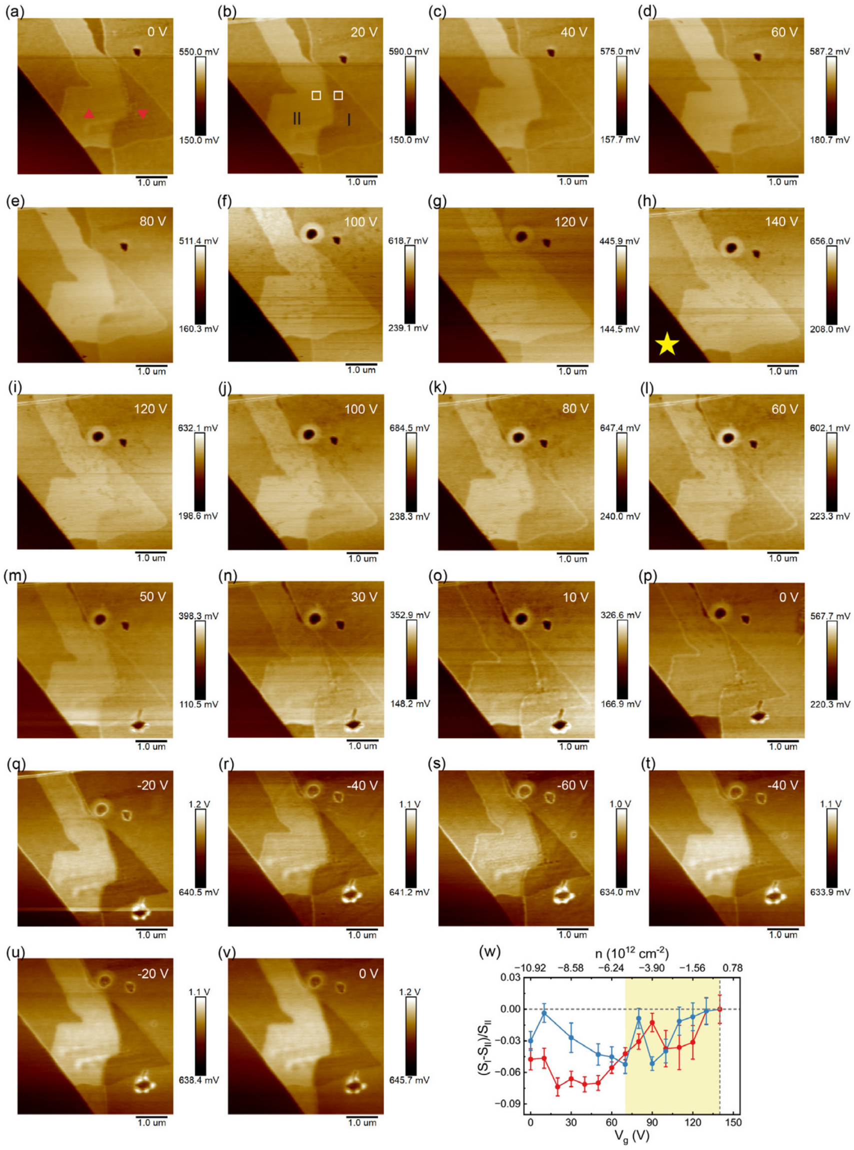

Figure S8. Full dataset for gate-dependent SNOM of device 2. (a)-(v) SNOM images at different V_g values following the applied V_g sequence in our experiment between -60 V and 140 V. The optical near-field response between the two adjacent polar states becomes almost identical at approximately V_g = 140 V (h), indicating strong hole doping in the sample at V_g = 0 V, which is presumably due to unintentional doping from the substrate. Owing to this unintentional hole doping, we can identify ABAC and ABCB stackings without gating, and electron doping of the sample is not achieved. (w) Optical contrast between two adjacent polar stackings as a function of V_g. Red and blue dots mark extracted optical contrasts measured from V_g = 0 V to the positive maximum V_g and the positive maximum V_g to the negative maximum V_g, respectively. The charge neutrality point roughly lies at V_g = 140 V (indicated by the gray dashed lines), confirmed by the vanishing amplitude of the plasmon fringes reflected by the DW. The yellow shaded area marks the region from a carrier density of 0 to a hole doping density of $5.46 \times 10^{12} cm^{-2}$, in which the optical contrast tends to increase with increasing hole doping. The white boxes in (b) label the sample areas from which the optical contrasts were extracted.

Supplementary Figure 9.

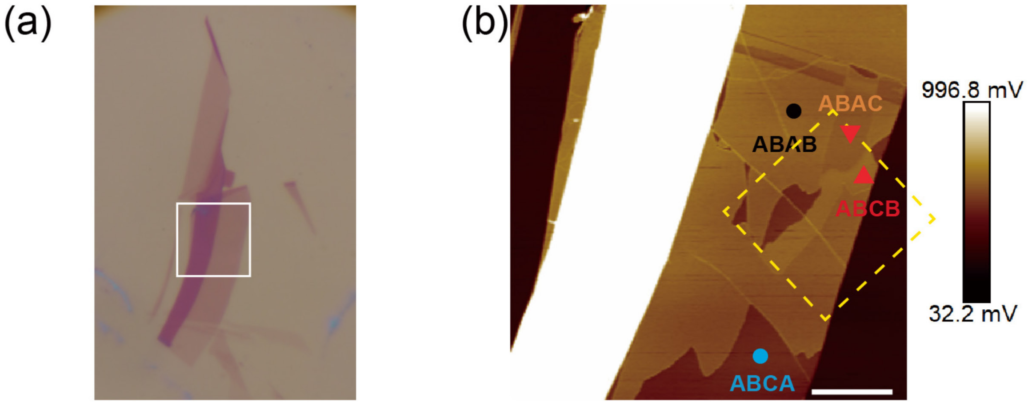

Figure S9. Raman spectra measurement of device 2. (a) Optical image of the tetralayer graphene. The white box marks the scanning area of (b). (b) SNOM image of the tetralayer graphene area shown inside the white box in (d), which contains four different optical near-field contrasts at $V_g = 0$ V. The yellow dashed frame labels the scanning area of KPFM mapping in Figure 4d. The different symbols in (b) mark the positions where the Raman spectra in Figure 4a were taken. Note that the small area of ABCA in the yellow dashed frame makes direct far-field Raman measurements challenging. Instead, we measure the Raman spectra of the nearby ABCA domain with identical optical near-field responses [blue dot in (b)]. The scale bar in (b) is 4 μm.

Supplementary Figure 10.

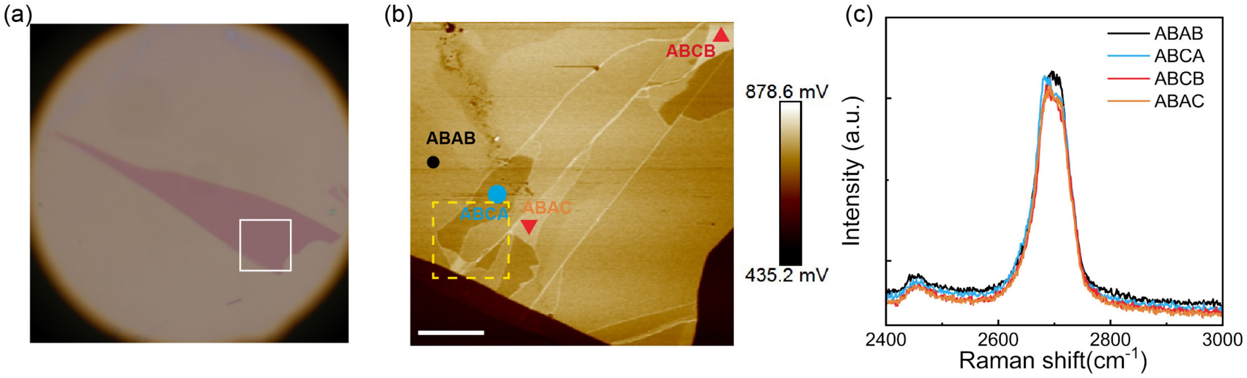

Figure S10. Raman spectra of device 3. (a) Optical image of the tetralayer graphene. The white box marks the scanning area of (b). (b) SNOM image of the same tetralayer graphene area shown inside the white box in (a), which contains four different optical near-field contrasts at $V_g = 0$ V. The yellow dashed frame labels the scanning area of KPFM mapping shown in Figure S11. (c) Raman spectra of the four different stackings. Different symbols in (b) mark the positions where the Raman spectra in (c) are taken. Note that the small area of polar domains [at the corner of the yellow dashed frame in (b)] makes direct far-field Raman measurements challenging. We instead measure the Raman spectra of the nearby polar domain that shows an identical optical near-field response [red triangles in (b)]. The scale bar in (b) is 4 µm.

Supplementary Figure 11.

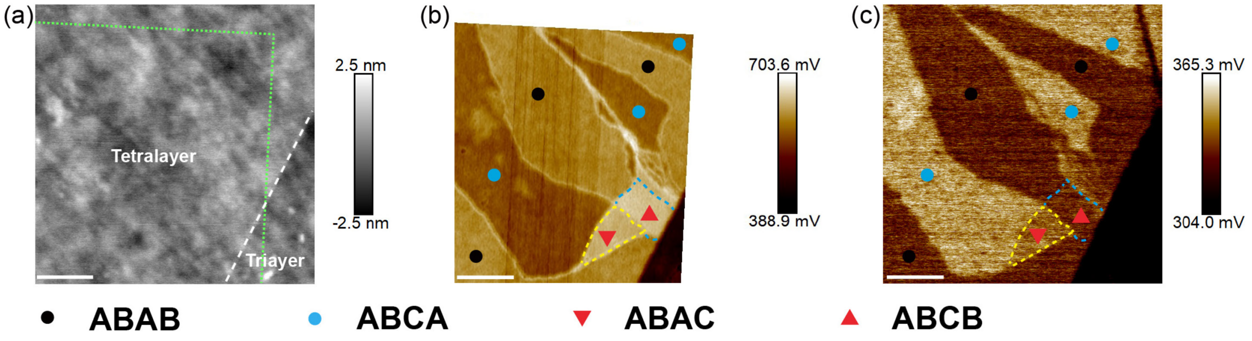

Figure S11. KPFM measurement of device 3 at $V_g = 0$ V. (a) Topography of the tetralayer graphene. (b) SNOM image of the green dashed area in (a). Domains with distinct stackings are labeled by different symbols in color. At $V_g = 0$ V, the two polar stackings (indicated by triangles) show different near-field optical contrast, since this sample is hole-doped at $V_g = 0$ V. The stacking assignments in (b) are also supported by our Raman spectra in Figure S10 and the KPFM result in (c). (c) KPFM mapping of the same area as in (a) at $V_g = 0$ V. For the two nearby polar stackings, the one that is darker (brighter) in SNOM image appears to be brighter (darker) in KPFM, indicative of the downward polarization [downward triangle in (b) and (c)] and upward polarizations [upward triangle in (b) and (c)], respectively. The blue dashed line marks the boundary of the ABCB domain, and the yellow dashed line marks the boundary of the ABAC domain. The KPFM results of device 3 are consistent with those of device 1 (Figure 2i-k), device 2 (Figure 4b-d), and device 4 (Figure S14). All the scale bars are 1 µm.

Supplementary Figure 12.

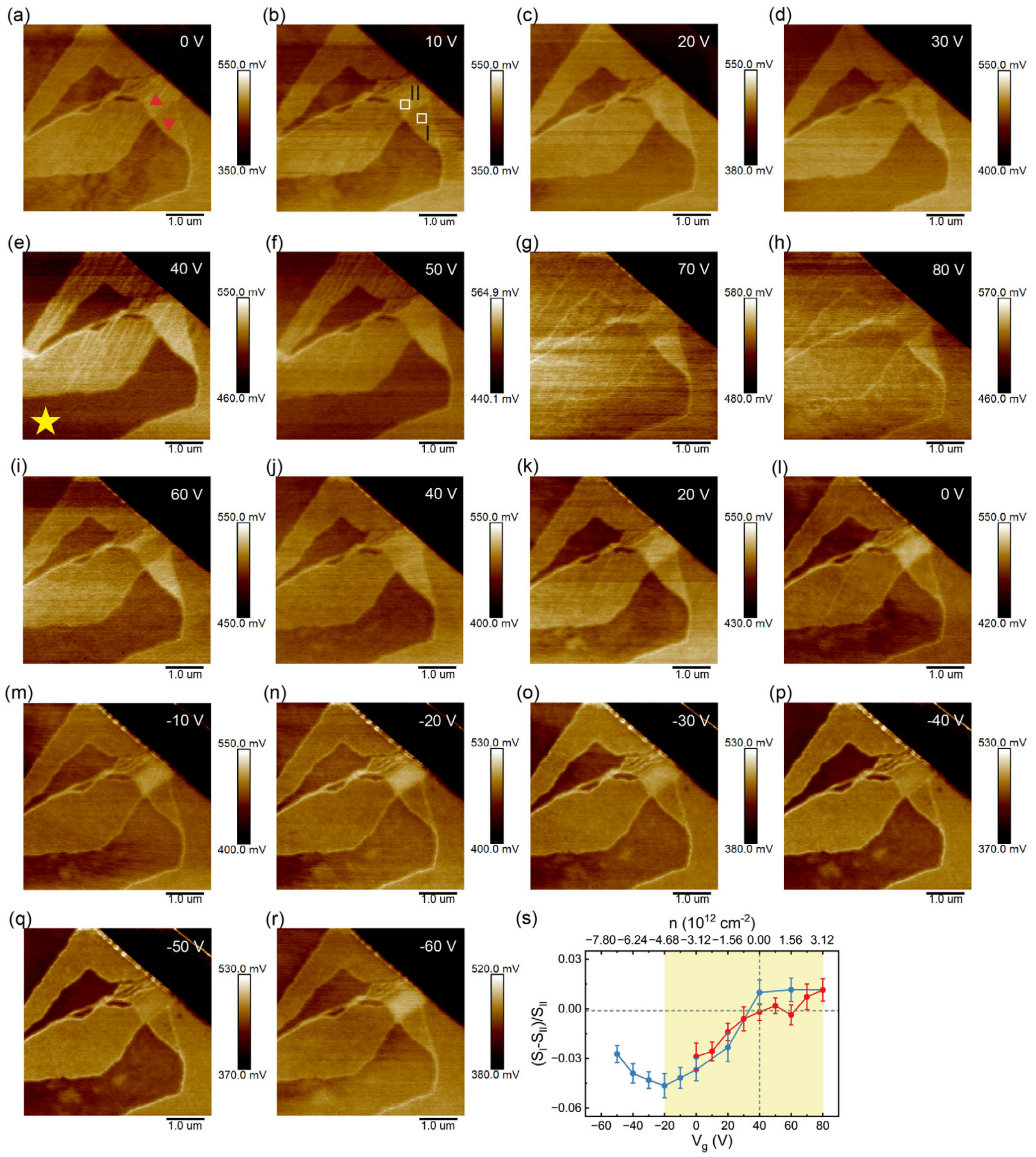

Figure S12. Full dataset for the gate-dependent SNOM of device 3. (a)-(r) SNOM images at different V_g following the sequence of applied V_g in our experiment between -60 V and 80 V. The optical near-field response between the two adjacent polar states becomes almost identical at around V_g = 40 V (e), indicating hole doping in the sample at V_g = 0 V. This means that the sample is slightly hole-doped at V_g = 0 V, which prevents our tetralayer from being further electron-doped. (s) Optical contrast between the two adjacent polar stackings, as a function of V_g. Red/blue dots mark extracted optical contrasts measured from V_g = 0 V to the positive maximum V_g and the positive maximum V_g to the negative maximum V_g, respectively. The charge neutrality point roughly lies at V_g = 40 V (indicated by the gray dashed lines), confirmed by the vanishing amplitude of the plasmon fringes reflected by the DW. The yellow shaded area marks the carrier density from electron doping density of $\mathbf{3.12 \times 10^{12} cm^{-2}}$ to hole doping density of $\mathbf{4.68 \times 10^{12} cm^{-2}}$, where the optical contrast shows an increased trend with increasing doping. The white boxes in (b) label the sample areas in which the optical contrasts are extracted.

Supplementary Figure 13.

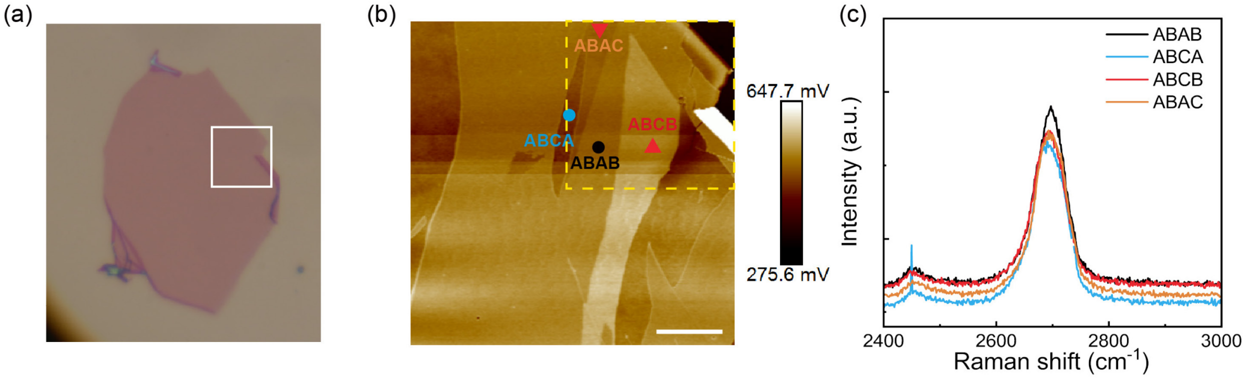

Figure S13. Raman spectra of device 4. (a) Optical image of the tetralayer graphene. The white box marks the scanning area of (b). (b) SNOM image of the same tetralayer graphene area shown inside the white box in (a), which contains four different optical near-field contrasts at $V_g = 0$ V. The yellow dashed frame labels the scanning area of KPFM mapping shown in Figure S14. (c) Raman spectra of the four different stackings. Different symbols in (b) mark the positions where the Raman spectra in (c) are taken. To better distinguish the spectra of different stackings, we manually shift the spectrum of ABCA stacking. The scale bar in (b) is 6 µm.

Supplementary Figure 14.

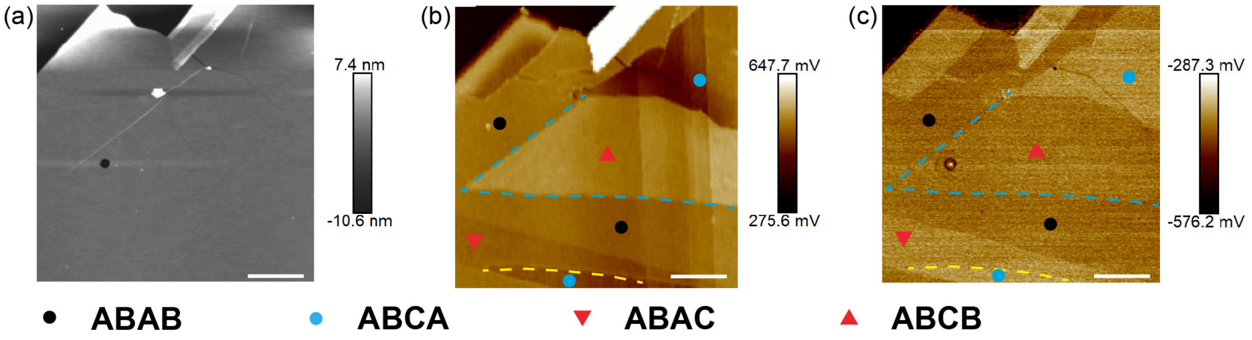

Figure S14. KPFM measurement of device 4 at $V_g = 0$ V. (a) Topography of the tetralayer graphene. (b) SNOM image of the same area as in (a). Domains with distinct stackings are labeled by different symbols in color. At $V_g = 0$ V, the two polar stackings (indicated by triangles) show different near-field optical contrast, since this sample is hole-doped at $V_g = 0$ V. The stacking assignments in (b) are also supported by our Raman spectra in Figure S13 and the KPFM result in (c). (c) KPFM mapping of the same area at $V_g = 0$ V. For the two nearby (not directly being adjacent) polar stackings, the one that is darker (brighter) in SNOM image appears to be brighter (darker) in KPFM, indicative of the downward polarization [downward triangle in (b) and (c)] and upward polarizations [upward triangle in (b) and (c)], respectively. The blue (yellow) dashed line marks the boundary between the ABAB domain and the ABCB domain (between the ABCA domain and the ABAC domain), respectively. The KPFM results of device 3 are consistent with those of device 1 (Figure 2i-k), device 2 (Figure 4b-d), and device 3 (Figure S11). All the scale bars in (a), (b), and (c) are 3 µm.

Supplementary Figure 15.

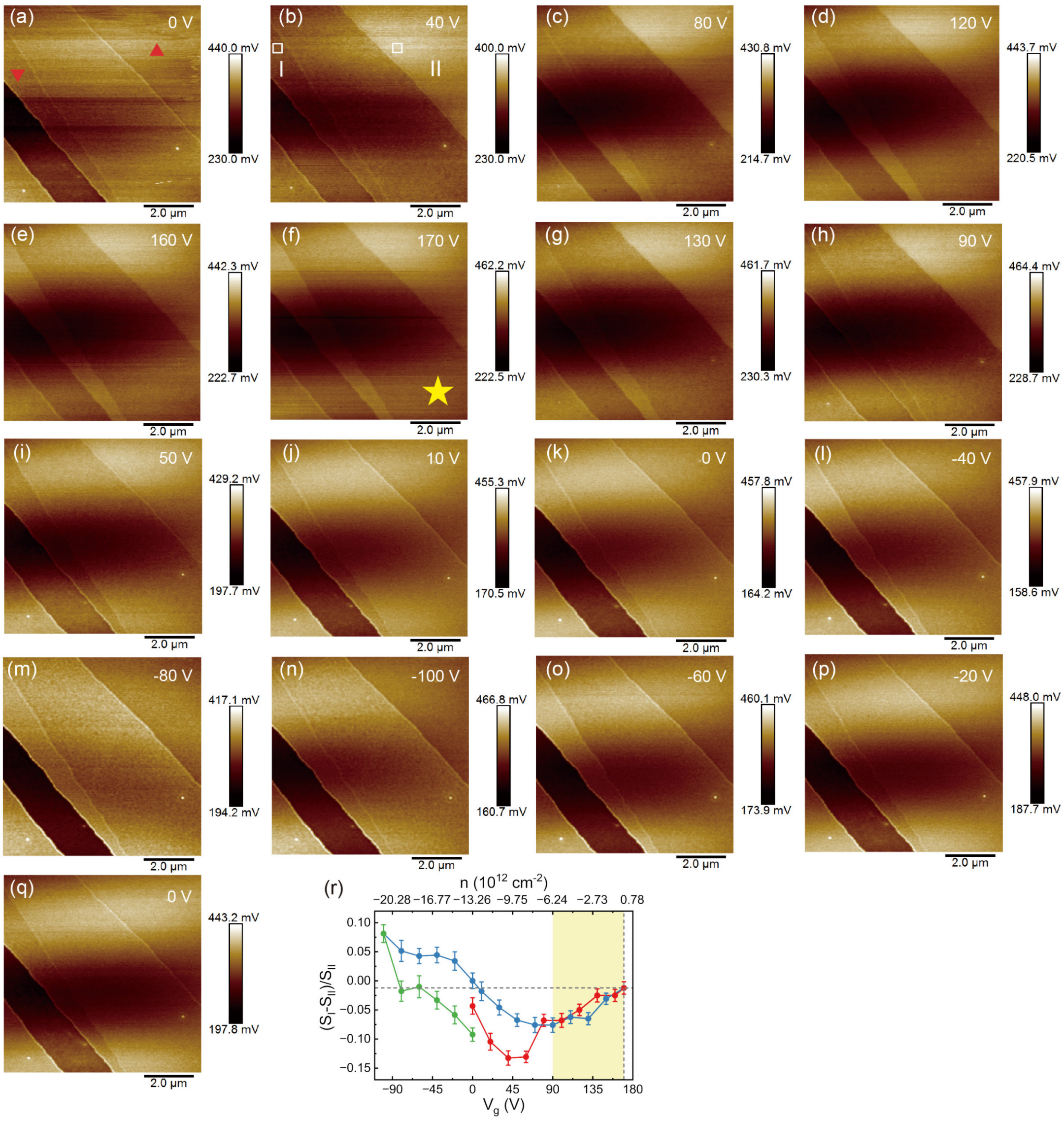

Figure S15. Full dataset for the gate-dependent SNOM of device 4. (a)-(q) SNOM images at different V_g following the sequence of applied V_g in our experiment, between -100 V and 170 V. The optical near-field response between the two nearby polar states becomes almost identical at around V_g = 170 V (f), indicating a strong hole doping at V_g = 0 V, which prevents our tetralayer from being electron-doped. We note that fringe features appearing in the vertical direction of SNOM images are presumably due to the interference effect caused by the scattered light from the nearby silver paste used to make sample contact. (r) Optical contrast between the two nearby polar stackings, as a function of V_g. Red/blue/green dots mark optical contrasts along V_g = 0 V to positive maximum V_g, positive maximum V_g to negative maximum V_g, and negative maximum V_g to V_g = 0 V trajectories, respectively. The charge neutrality point roughly lies at = 170 V (indicated by the gray dashed lines), confirmed by the vanishing amplitude of the plasmon fringes reflected by the DW. The yellow shaded area marks the carrier density from 0 to hole doping density of $\mathbf{6.24} \times \mathbf{10^{12}} \boldsymbol{cm^{-2}}$, where the optical contrast shows an increasing trend with increasing hole doping. The white boxes in (b) label the sample areas in which the optical contrasts are extracted.

Supplementary Figure 16.

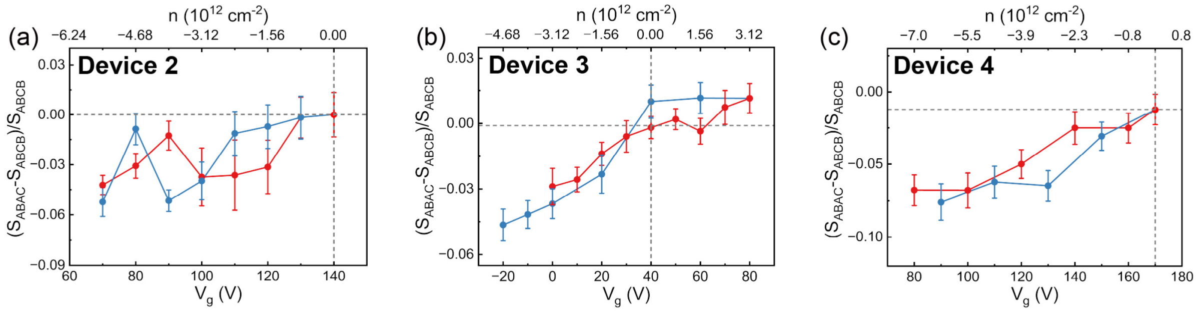

Figure S16. Extracted optical contrast between the two polar stackings of devices 2-4 (within a certain carrier density range). (a)-(c) Optical contrasts between two adjacent polar stackings of devices 2-4 as a function of V_g (carrier density n). The optical contrast in devices 2-4 tends to increase with increasing doping, corresponding to the yellow shaded areas in Figure S8w, Figure S12s, and Figure S15r. The dashed lines in (a) to (c) indicate the charge neutrality points in the three devices. The gate-dependent optical contrasts for the forward scan (increasing V_g) and backward scan (decreasing V_g) are denoted by the red and blue dots, respectively.

Supplementary Figure 17.

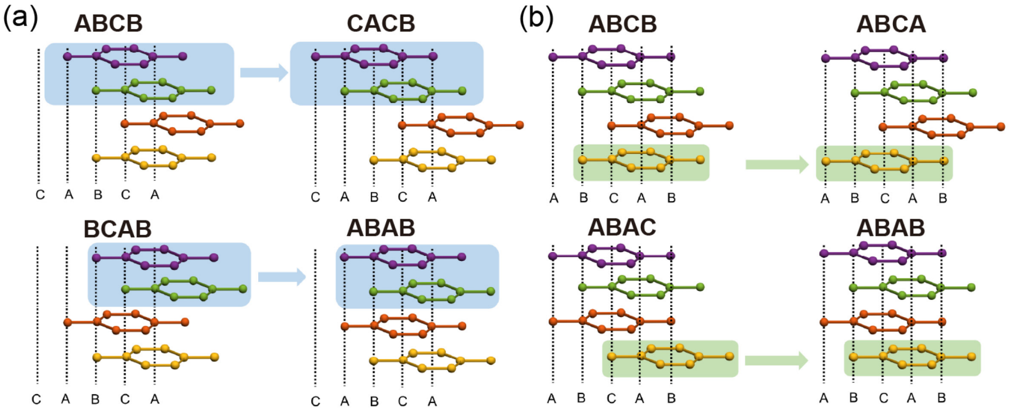

Figure S17. Schematic illustration of a second possibility regarding the interlayer sliding. (a) Schematics of the second possibility, in which sliding the top two layers together also corresponds to the blue DW motion in Figure 3f. Note that ABCB and CABA, and BCAB and ABCA stackings are equivalent stacking orders. (b) Schematics of the second possibility, in which sliding the bottom layer only also corresponds to the green DW motion in Figure 3f. Note that ABCB and CABA, and ABAC and CACB stackings are equivalent stacking orders.

Supplementary Figure 18.

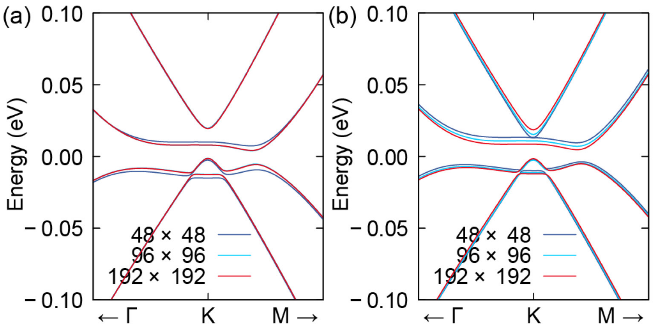

Figure S18. Convergence of the band structures against k-point mesh numbers. (a) Band structures obtained with half-grid shift. (b) Band structures obtained without shift. We observe that the results with $\boldsymbol{N} = \mathbf{96}$ and $\boldsymbol{N} = \mathbf{192}$ overlap within the line thickness in (a), while convergence is slow in (b).
